# Reliability, reference values and factors related to maximum bite force measured by the Innobyte system in healthy adults with natural dentitions

**DOI:** 10.1007/s00784-024-06014-5

**Published:** 2024-10-31

**Authors:** Mireia Ustrell-Barral, Carla Zamora-Olave, Laura Khoury-Ribas, Bernat Rovira-Lastra, Jordi Martinez-Gomis

**Affiliations:** 1 Department of Prosthodontics, School of Dentistry, Faculty of Medicine and Health Sciences, University of Barcelona. C/ Feixa Llarga S/N, L’Hospitalet de Llobregat, 08907 Barcelona, Catalonia Spain; 2 Oral Health and Masticatory System Group (Bellvitge Biomedical Research Institute) IDIBELL. L’Hospitalet de Llobregat, Barcelona, Catalonia Spain

**Keywords:** Bite force, Bruxism, Overbite, Handgrip strength, Reliability, Reference values

## Abstract

**Objectives:**

We aimed to determine the predictors of maximum bite force (MBF), as measured with the Innobyte system, and to assess the reliability and reference values for MBF in young adults with natural dentitions.

**Methods:**

This cross-sectional test–retest study included 101 dental students with natural dentitions. Participants had their dental occlusion examined and completed three questionnaires: the Temporomandibular disorders Pain Screener, Oral Behavior Checklist, and Jaw Functional Limitation Scale. Body mass index and muscle mass percentage were determined, and handgrip strength was measured with a dynamometer. The MBF was measured with Innobyte, with reliability assessed by the intraclass correlation coefficient, expressing reference values as MBF percentiles. Bivariate tests and multiple linear regression models were used for statistical analysis.

**Results:**

The intraclass correlation coefficient for the MBF was 0.90, with 10th to 90th percentiles of 487–876 N for females and 529–1003 N for males. A positive relationship existed between the MBF and male sex, muscle mass percentage, overbite, handgrip strength, and possible sleep/awake bruxism. Stepwise regression showed that overbite, handgrip strength, and possible sleep/awake bruxism had the greatest effect on the MBF, explaining 27% of the variation.

**Conclusions:**

This study provides reference values for MBF when using the Innobyte system and shows excellent reliability. Overbite, general strength, and self-reported bruxism appear to be important predictors of MBF.

**Clinical relevance:**

Innobyte is a reliable device that can be used to measure MBF bilaterally. Self-reported bruxism is associated with an 8%–10% increase in MBF.

## Introduction

The maximum bite force (MBF) is a key voluntary factor that affects masticatory performance [1]. As such, it has been used to measure masticatory function objectively [2], assess improvement after prosthodontic treatment [3, 4], and describe the manifestations of musculoskeletal and/or neurodegenerative diseases [5–7]. Several factors affect the MBF, including age, gender, nutritional status, general strength, dental status, craniofacial morphology, dental occlusion, temporomandibular disorders (TMD), and bruxism [7, 8]. The MBF in humans is greatest between the ages of 20 and 45 years [8, 9]. Most studies have reported that males have higher MBF values than females [1, 10–13], though some studies have found no differences [14, 15]. Although nutritional status is thought to influence MBF, studies have found no association between body mass index (BMI) and MBF, probably because BMI does not reliably link to skeletal muscle mass [10, 11, 16]. General strength, normally measured by handgrip strength using a dynamometer, correlates highly with the MBF [17, 18]. Individuals with natural dentitions also have an MBF that is 5 to 7 times greater than in the edentulous, although the prosthesis used affects the magnitude of this difference [3, 18]. Craniofacial morphology and occlusal characteristics have been associated with MBF [9, 10, 15, 19–24], but existing studies have not produced consistent results. Concerning TMD pain, MBF may be lower in affected patients than in controls and improve with treatment [25–28], thought findings in the general population indicate similar bite forces in individuals with and without signs and symptoms of TMD [10, 11]. The relationship between MBF and bruxism or other oral behaviors is unclear. One study has reported that females with TMD and bruxism had a reduced MBF [29], whereas two studies have found that individuals with bruxism had higher MBF than non-bruxists [30, 31]. However, most studies have found no association [11, 15, 32–34].

Several aspects of bite force measurement can affect MBF values. Bilateral clenching has been shown to produce an MBF about 30–40% higher than unilateral [9, 35]. When measured unilaterally, the force obtained in the region of the first molar is 1.4 and 3.2 times higher than that obtained in the first premolar and incisor regions, respectively [1]. Moreover, the force on the preferred chewing side is higher than on the non-preferred side [36, 37]. The device can also affect the MBF, with clenching on soft surfaces and reduced vertical separation of the jaws known to increase MBF values [12, 38]. Recently, Kube Innovation (Montreal, Quebec, Canada) developed the Innobyte device to measure bite force through bilateral clenching. This device comprises a soft silicone bite fork that compresses a volume and provides a pressure value in Newtons on an LCD [4]. Although this system is ease to use, comfortable for patients, and more than 95% accurate [4, 39, 40], its reliability when measuring the MBF is not known. In addition, the manufacturer provides a graphic with MBF reference values, considering values > 1000 N excessive, values 650–1000 N normal, and values < 650 N to indicate a deficit. However, neither the target population nor the methodology used to obtain these references are reported, leaving scope for improvement in two aspects. First, reference values should be reported for males and females, reflecting that most studies suggest a gender dimorphism in bite force [1, 10, 11, 16]. Second, although it is usual to perform three or more measurements, it is not known whether to use the highest value, the mean value, or the average of the two highest values from the three or more trials.

The primary aim of this study was to identify factors explain MBF variability measured with the Innobyte system in young healthy adults with natural dentitions. The secondary aims were to assess the test–retest reliability, to establish the reference values, and to explore the most reliable way to assess MBF using the Innobyte system.

## Materials and methods

### Study design and population

This cross-sectional test–retest study included 107 dental students from the University of Barcelona, aged 18–45 years, with a minimum of 24 natural teeth and no edentulous spaces. Those with neurological disorders, taking muscle-relaxant or sedative-hypnotic medications, dental prostheses, severe malocclusion (negative overjet, negative overbite, midline deviation greater than 4 mm, or presence of scissor bite), periodontal disease, or active orthodontic treatment with fixed appliances were excluded. All participants signed the written informed consent form approved by the Ethics Committee of Barcelona University Dental Hospital (Ref. 29/2023). All procedures were conducted according to the principles of the Helsinki Declaration. Reporting follows the Strengthening the Reporting of Observational Studies in Epidemiology (STROBE) guidelines.

## Procedure and data collection

Participants were interviewed to collect data on age, gender, and history of orthodontic treatment. They also completed three questionnaires: the TMD Pain Screener (TMD-PS) to detect pain related to TMD [41], the Oral Behavior Checklist (OBC) to assess the frequency of oral behaviors [42, 43], and the 20-item Jaw Functional Limitation Scale (JFLS) to quantify any functional limitation of the masticatory system [44, 45]. A new variable named “possible sleep/awake bruxism” was created by summing the results of the first, third, and fourth questions of the OBC questionnaire that relate to the frequency of clenching and/or grinding the teeth [46]. Results were dichotomized using the median as a cutoff [43, 47]: scores of 0–3 indicated “non- or mild-bruxers” while scores of 4–12 indicated “moderate-to-frequent bruxers” [48]. Similarly, results from the questionnaires TMD-PS and JFLS were also dichotomized. For the TMD-PS, scores of 0 indicated “no self-reported TMD pain” and scores of ≥ 1 indicated “self-reported TMD pain.” For the JFLS, scores of 0 indicated “no jaw functional limitation” and scores of ≥ 1 indicated “jaw functional limitation.”

Various clinical examinations were also conducted. Dental examination recorded the molar Angle class bilaterally and measured midline deviation, overjet, and overbite by caliper [49]. Body weight and height were measured with the individuals barefoot and without excess/heavy clothing. The BMI was calculated as weight divided by height squared (kg/m^2^) [50]. Upper arm circumference (UAC) was measured twice at the midpoint between the olecranon and acromion of both arms with a non-stretchable UAC-tape (Lufkin W606PM, Crescent Tools, Sparks, MD, USA). Triceps skinfold thickness (TSF) was measured twice at the back of both arms using a Slim Guide skinfold caliper (Creative Health Products, Ann Arbor, MI, USA) with a static pressure of 10 g/mm^2^ in the middle of the triceps: the examiner gently pinched skin away from the underlying muscle and measure TSF with the caliper. The UAC and TSF measurements were performed by the same examiner, at the same time of the day, with the participant seated and the arms relaxed and extended freely. Average UAC and TSF measurements were used to calculate the arm muscle area (AMA), the arm area (AA), and the muscle mass percentage (MM%), according to the following equations [51].

$$\text{AMA }\left({\text{cm}}^{2}\right)= \left(\text{UAC}-\uppi \times \text{TSF}\right) 2/{4}_{\uppi }$$

$$\text{AA}= {\left(\text{UAC}/\uppi \right)}^{2} \times\uppi /4$$

$$\mathrm{MM}\%\;=\;\mathrm{AMA}\;\times\;100/\mathrm{AA}$$

Handgrip strength in kilograms was measured three times in each hand (right, left, left, right, right, left) using a hand-held grip dynamometer (EH-101, Camry Scale, South El Monte, CA, USA) with rest periods of up to 60 s after measurements. Participants were seated on a chair in front of a table on which they supported their forearm in a supinated position and their elbow in 90º flexion. They were asked to squeeze the dynamometer at maximum force for 3 s, while the investigator held the device and recorded the maximum value.

Bilateral bite force was measured using the Innobyte system (Kube Innovations, Montreal), according to the manufacturer instructions. With the participant seated and the head supported in a comfortable position, an examiner inserted the mouthpiece with a new disposable cover into the individual’s mouth, placing their upper central incisors against the protruding stop at the front of the mouthpiece; cheek guards were also placed against their molars. The participant was asked to close slowly to ensure that the mouthpiece was placed correctly between the maxillary and mandibular arches, and once confirmed, the participant was instructed to bite on the mouthpiece with maximum effort (for 1–3 s). The bite force, displayed in Newtons on the screen, was recorded. Three measurements were performed, with participants allowed a resting period of 10 to 60 s between measurements.

## Data analysis

Sample size was calculated based on the common rule of thumb that a ratio of 10 participants per independent variable is required for multiple regression analysis [52]. Since we designed this study to assess the relationship of 10 independent variables, with the MBF as the dependent variable, we required 100 participants.

To determine test–retest reliability, all participants were invited to repeat the handgrip strength and bite force measurements 2 weeks after the first session. Reliability was assessed by intraclass correlation coefficient (ICC) for average measurements, using a two-way random effects model and absolute agreement. The smallest detectable difference (SDD) was determined as the smallest statistically significant amount of change that could be detected with a measurement device on two different occasions, and it was calculated as follows.

$$\text{SDD}=1.96 \times \sqrt{2 \times \text{SEM}}$$

The Kolmogorov–Smirnov was used to confirm the normality of the distribution for bite force variables. MBF percentiles (10th, 25th, 50th, 75th, 90th) were calculated by gender and assessment [53, 54]. A general linear model with repeated measures was used to assess the effects of two within-individual factors (session, trial) and one between-individual factor (gender) on the variance of MBF as a dependent variable. Bivariate associations between variables (i.e., gender, age, BMI, muscle mass percentage, history of orthodontic treatment, bilateral Angle class I, overbite, overjet, midline deviation, handgrip strength, oral behavior, possible sleep/awake bruxism, self-reported TMD pain, and jaw limitation) and MBF assessed as average of the top two values were evaluated using Pearson correlation, Spearman correlation, or student t-tests. Finally, because some independent variables were interrelated, a multiple linear regression model was performed, using a stepwise forward method to examine whether those variables significantly associated with MBF contributed meaningfully to explaining the variance in MBF. All data were analyzed in IBM SPSS, version 29 (IBM Corp., Armonk, NY, USA) and p-values below 0.05 were considered significant.

## Results

Among the 107 dental students invited to participate in this study, three did not meet the inclusion criteria (2 receiving active orthodontic treatment with fixed appliances and 1 with < 24 natural teeth), and 2 did not accept the invite. Another woman reported pain in the maxillary right central incisor due to a history of dental trauma and did not perform an MBF, so was excluded from the study. Therefore, data from 101 participants were included in the analysis. Among these, partial data were missing for several participants: two females asked not to have their weight measured; three females did not answer questions about orthodontic treatment; one woman did not perform left hand grip strength measurement because she had injured some fingers of that hand; and one man and one woman did not complete the TMD Pain Screener, OBC, or JFLS questionnaires. In the retest session, one man did not attend, one woman did not perform the bite force measurements, and another woman did not perform the handgrip strength measurement because of injury to the fingers of their right hand. During the bite force measurements, three individuals reported some pain or discomfort in the temporomandibular joint and one reported some pain or discomfort in the masticatory muscles.

The population characteristics are summarized in Table 1. The mean age was 22.8 years (range, 19.9 – 40.4) and most were female 88 (87%). Males showed higher handgrip strength, bite force, and muscle mass percentage than females, but we observed no gender differences in occlusal characteristics, oral behavior frequency, self-reported TMD pain, or jaw functional limitation. Table 2 shows the ICC and SDD values for handgrip strength and MBF by the assessment method. Mean handgrip strength and the average of the top two MBF results provided the highest ICC and lowest SDD values. In general, reliability was higher when measuring handgrip strength than MBF, regardless of the assessment method (Table 2).

**Table 1:** Population description by gender

	N	Total	Females (n = 88)	Men (n = 13)	Significance (P)
Age (years)	101	22.8 (3.5)	22.7 (3.1)	24.0 (5.6)	0.494^a^
Weight (kg)	99	64.1 (11)	62.4 (11)	75.7 (10)	< 0.001^a^
Height (m)	101	1.67 (0.07)	1.66 (0.06)	1.76 (0.06)	< 0.001^b^
Body mass index (kg/m^2^)	99	22.8 (3.3)	22.6 (3.4)	24.2 (2.3)	0.091^b^
Upper arm circumference (mm)	101	269 (34)	264 (31)	308 (23)	< 0.001^a^
Triceps skinfold (mm)	101	18.4 (6.7)	18.7 (6.9)	16.6 (4.7)	0.411^a^
Upper arm area (mm^2^)	101	5869 (1496)	5618 (1387)	7573 (1051)	< 0.001^a^
Muscle mass percentage (%)	101	62.5 (9.2)	61.5 (9.1)	69.4 (7.1)	0.003^b^
History of orthodontic treatment	98	68.4%	71.8%	46.2%	0.064^c^
Bilateral Angle Class I	100	91.0%	90.8%	92.3%	0.860^c^
Overbite (mm)	101	2.7 (1.3)	2.6 (1.3)	3.3 (1.2)	0.170^a^
Overjet (mm)	101	2.7 (1.1)	2.7 (1.1)	2.5 (1.1)	0.144^a^
Midline deviation (mm)	101	0.8 (0.9)	0.8 (0.7)	1.4 (1.3)	0.070^a^
Handgrip strength (kg)					
Highest value	101	32.5 (7.8)	30.4 (5.2)	47.0 (6.7)	< 0.001^b^
Average top 2 values	101	31.8 (7.6)	29.7 (5.1)	46.1 (6.5)	< 0.001^b^
Average maximum right/left values	100	30.8 (7.6)	28.7 (5.0)	44.8 (6.6)	< 0.001^b^
Mean value	101	29.2 (7.2)	27.3 (4.9)	42.5 (6.3)	< 0.001^b^
Oral behavior checklist (sum score)	99	20.9 (8.4)	20.9 (8.5)	20.7 (8.3)	0.937^b^
Possible sleep/awake bruxism	99	43.4%	40.2%	66.7%	0.087^c^
Self-reported TMD Pain	99	49.5%	50.6%	41.7%	0.563^c^
Jaw Functional Limitation	99	46.5%	47.1%	41.7%	0.722^c^
Maximum bite force (N)					
Highest value	101	710 (153)	693 (147)	826 (154)	0.003^b^
Average top 2 values	101	687 (149)	673 (143)	782 (164)	0.013^b^
Mean value	101	660 (148)	647 (139)	748 (176)	0.020^b^

^a^Mann–Whitney *U* test, ^b^ student t-test, ^c^ chi-square test

TMD, temporomandibular disorder

**Table 2:** Test–retest reliability and measurement error of different muscular force variables assessed by different ways

Muscular force variable	n	ICC (95% CI)	*P* -value	SDD
Handgrip strength				
Highest value	100	0.976 (0.96–0.98)	< 0.001	0.67 kg
Average of top 2 values	100	0.978 (0.97–0.99)	< 0.001	0.64 kg
Average maximum right/left values	98	0.979 (0.97–0.99)	< 0.001	0.61 kg
Mean value	100	0.979 (0.97–0.99)	< 0.001	0.58 kg
Maximum bite force				
Highest value	99	0.895 (0.84–0.93)	< 0.001	27.2 N
Average of top 2 values	99	0.903 (0.85–0.94)	< 0.001	24.9 N
Mean value	99	0.889 (0.83–0.93)	< 0.001	26.4 N

ICC, 2-way random, absolute agreement for average measurements

SDD is expressed in kg for handgrip strength and in Newtons for maximum bite force

Abbreviations: CI, Confidence interval; ICC, intraclass correlation coefficient; SDD, smallest detectable difference

**Fig. 1:**
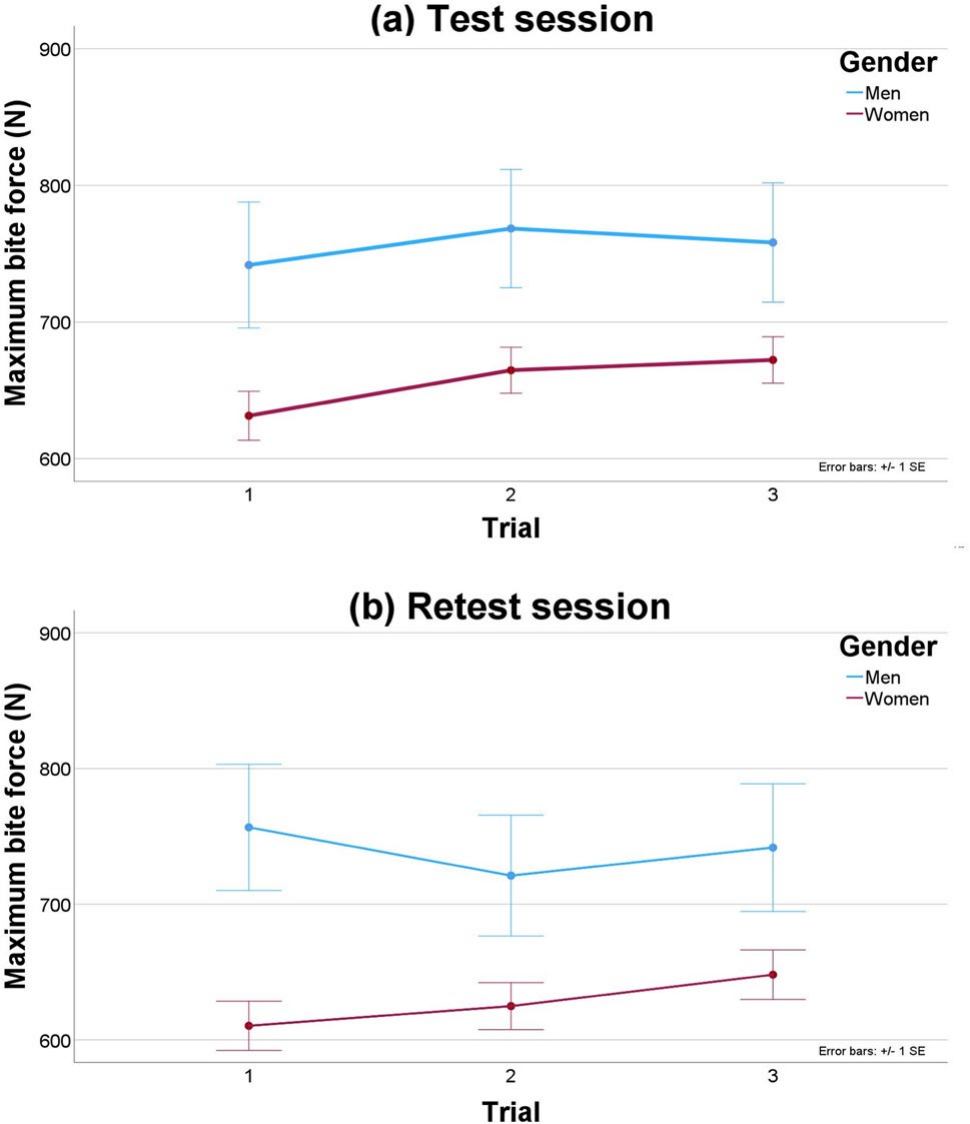
Plots of maximum bite force in the **a** test and **b** retest session by gender and by trial

The overall median MBF, assessed as the average of the top 2 measurements, was 675 N (range, 584–799 N and 496–884 N for the 25th–75th and 10th–90th percentiles, respectively). In females, this median MBF was 670 N (range, 580–773 N and 487–876 N for the 25th–75th and 10th–90th percentiles, respectively). By contrast, reference values were higher in males, with a median MBF of 807 N (range, 633–922 N and 529–1003 for the 25th–75th and 10th–90th percentiles, respectively) (Table 3). The repeated-measures general linear model showed that the MBF depended on gender (*p* < 0.014), but not on the session (test vs retest) (*p* = 0.118) or the trial (first, second, or third) (*p* = 0.176) (Fig. 1). However, an interaction was detected between gender and trial (*p* < 0.001), with females giving progressively higher MBF values on each trial.

**Table 3:** Maximum bite force percentiles by gender and by different ways of assessment

	Maximum bite force (N)					
Percentile	Females (n = 88)			Men (n = 13)		
	Highest value	Average top 2 values	Mean values	Highest value	Average top 2 values	Mean values
Minimum	329	322	316	581	517	448
10th	493	487	473	588	529	478
25th	598	580	532	664	633	590
50th	687	670	651	884	807	764
75th	790	773	740	949	922	906
90th	894	876	861	1017	1003	978
Maximum	1005	964	943	1029	1019	1002

Males and moderate-to-frequent bruxers had higher MBF values than females and non- or mild-bruxers (Table 4). Muscle mass percentage, overbite, and handgrip strength were positively associated with MBF. Stepwise regression analysis showed that overbite, handgrip strength, and possible sleep/awake bruxism were the most important factors affecting MBF when assessed as the average of the highest two values (Table 5). These three variables accounted for 27% of the variation in MBF (adjusted R^2^ = 0.27).

**Table 4:** Bivariate relationship between the different factors and the maximum bite force assessed as average of top 2 values

	n	Correlation with MBF (N)	Mean difference of MBF (N) between groups (95% CI)	Significance (*P*)
Gender (men vs females)	101		109 (23 to 195)^c^	0.013
Age (years)	101	0.03^b^		0.741
Body mass index (kg/m^2^)	99	0.01^a^		0.931
Muscle mass percentage (%)	101	0.25^a^		0.011
History of orthodontic treatment	98		50 (-15 to 115)^c^	0.132
Bilateral Angle class I (yes vs no)	100		0.52 (-104 to 105)^c^	0.992
Overbite (mm)	101	0.43^a^		< 0.001
Overjet (mm)	101	0.13^b^		0.180
Midline deviation (mm)	101	-0.04^b^		0.724
Handgrip Strength (kg)	101	0.31^a^		0.002
Oral behavior checklist (sum score)	99	0.06^a^		0.576
Possible sleep/awake bruxism (yes vs no)	99		66 (7 to 125)^c^	0.028
Self-reported TMD Pain (yes vs no)	99		5 (-54 to 65)^c^	0.862
Jaw functional limitation (yes vs no)	99		-42 (-101 to 17)^c^	0.163

^a^Pearson coefficient

^b^Spearman rho coefficient

^c^student t-Test. Abbreviations: CI, confidence interval; MBF maximum bite force; TMD, temporomandibular disorder

**Table 5:** Forward stepwise regression model of factors related to maximum bite force (N) assessed as average of the top 2 values as a dependent variable

Step	Variables included	Unstandardized B (95%CI)	Significance (P)	R	R_a_^2^	F (Sig.)	F Change (Sig.)
1				0.43	0.18	22.4 (< 0.001)	22.4 (< 0.001)
	(Constant)	551 (489 to 613)	< 0.001				
	Overbite (mm)	50.4 (29 to 72)	< 0.001				
2				0.50	0.23	15.7 (< 0.001)	7.5 (0.007)
	(Constant)	417 (303 to 531)	< 0.001				
	Overbite (mm)	45.1 (24 to 66)	< 0.001				
	Handgrip strength (kg)	5.1 (1.4 to 8.7)	0.007				
3				0.54	0.27	13.1 (< 0.001)	6.2 (0.015)
	(Constant)	397 (285 to 510)	< 0.001				
	Overbite (mm)	46.4 (26 to 67)	< 0.001				
	Handgrip strength (kg)	4.7 (1.1 to 8.2)	0.011				
	Possible sleep/awake bruxism (no/yes)	64.4 (13 to 116)	0.015				

N = 99; Missing values Excluded cases listwise; Stepping criteria: 0.05 probability of F for entry, 0.010 for removal

R_a_^2^, adjusted R square (% variance explained); F (Sig.), F-value and significance

## Discussion

The results of this study indicate that overbite and general strength are the main predictors of MBF in young adults with natural dentitions. These factors explain 23% of the observed variation in MBF, while self-report bruxism explains an additional 4%. Gender and muscle mass percentage were both significantly correlated with MBF, but this correlation became insignificant after controlling for handgrip strength in the stepwise multiple regression analysis. Therefore, general strength seems to be more directly related to MBF than either gender or muscle mass percentage. As expected, the higher the general strength the higher the MBF, with a similar correlation coefficient (r = 0.30) to that reported in elderly females living in the community [17]; however, this was notably lower than the correlation reported in a recent study [18].

Other studies have found that individuals with short face heights (brachyfacial) or a short gonial angle have higher MBF values than those with long face heights (dolichofacial) or long gonial angles in bivariate or multivariate regression analyses [15, 19–24]. In the present study, overbite was the best predictor of MBF. Given that overbite is inversely related to facial height [55], it is plausible that individuals with higher force in their masticatory muscles during growth would develop more overbite and a lower facial height. Although this cross-sectional study found this positive and significant association between overbite and bite force in the multivariate model, we cannot demonstrate whether a large overbite is a cause or a result of MBF, or indeed, if there is a confounding factor that leads to a spurious relationship between overbite and bite force. Future longitudinal studies should be conducted to confirm the type of association between overbite and MBF.

The present results show that individuals who were self-rated themselves as moderate-to-frequent bruxers had an 8%–10% (or 64 N) higher MBF than those who rated themselves non- or mild-bruxers, after controlling for general strength and overbite. These results complement evidence that self-reported bruxers have an increased MBF in the incisor region [30] and that individuals with bruxism use higher bite forces for a given submaximal load than controls [56]. The higher MBF shown by individuals with bruxism could suggest a “training effect” if the increased muscular activity results in stronger muscles [57, 58]; indeed, in some individuals, bruxism could improve masticatory function. However, more research is needed to elucidate which type of bruxism (sleep vs awake, clenching vs grinding, and self-report vs clinical vs instrumental-based diagnoses) provides benefits, and on which aspects of masticatory function. This could be added to other putative benefits of bruxism, such as stress relief, preventing upper airway collapse in obstructive sleep apnea, increasing salivary flow, improving bone mineral density, and slowing cognitive decline [59].

The Innobyte system is reliable for measuring the bite force bilaterally, achieving similar ICC values to those in studies using gnathodynamometers or occlusal force gauges [1, 16, 18]. The SDD or measurement error was approximately 25 N, corresponding to less than 4% of the average MBF. These reliability and measurement error values are similar to, or even better than, those reported using a bite force transducer in children or adolescents [16, 60]. Among the different ways to assess MBF, using the average of the top 2 values achieved the highest reliability and lowest measurement error in this study, albeit with little difference. However, reliability and measurement error were significantly worse when measuring MBF than when measuring handgrip strength, probably due to the high intra-individual variability in clenching to obtain the MBF. This variability can be explained by several factors, such as concern about damaging teeth or the unpleasant sensation at the temporomandibular joint during measurement [9, 58]. Results of the general linear model analysis suggest that females, but not males, produce increasingly higher MBF values in the consecutive trials, probably due to being more prudent than males and only increasing force as they felt more confident. This could explain why the top two values could be the best way to assess the MBF in our cohort.

Since the Innobyte system allows the bilateral measurement of MBF, using all teeth, the fear of damage and inhibition by periodontal receptors can be reduced and a stronger force measured [12, 35, 61]. Therefore, MBF reference values measured with the Innobyte system are notably higher than those obtained with other devices [1, 15, 16, 18, 35]. Another advantage of the Innobyte system is that few individuals complained of pain or discomfort while clenching the soft blue silicon of the bite fork.

The present study, using the Innobyte system, suggests that normal MBF values in young adults with natural dentitions ranged from 510 to 940 N (median 750 N) when assessed as the average of the top two values from three measurements. These values should be established by gender if more precision is needed: 490 to 880 N (median 670 N) for females and 530 to 1000 N (median 810 N) for males. When interpreting MBF values measured using the Innobyte system, clinicians might consider these reference values to assess masticatory function indirectly [2], to evaluate improvements in prosthodontic treatment [3, 4], or to record manifestations of musculoskeletal or neurological disorders [5, 6, 62].

The main strength of this study is the sample size for the test–retest design. However, several important limitations require consideration. First, the inclusion of very few males means that the overall and male-specific reference values ​​should be interpreted with caution. Second, participants were recruited as a convenience sample of dental students and might not be representative of the whole population. Third, bruxism was only assessed by participant self-report and dichotomized; future studies should evaluate whether bruxism, when based on clinical findings and/or instrumentally assessed in a quantitative manner, remains associated with MBF.

## Conclusions

In young adults with natural and healthy dentitions, the variables overbite, general strength, and self-report bruxism most closely relate to the MBF. The Innobyte system offers greatest reliability for MBF measures when it uses the average of the top two values from three measurements, offering a measurement error of 25 N. The median (10th–90th percentile) reference values for MBF among young female and male adults with natural dentitions, as measured by the Innobyte system, are 670 N (490–880 N) and 810 N (530–1000 N), respectively.

## Data Availability

No datasets were generated or analysed during the current study.
